# Influenza and the Origins of The Phillips Collection, Washington, DC

**DOI:** 10.3201/eid1201.AD-1201

**Published:** 2006-01

**Authors:** David M. Morens, Jeffery K. Taubenberger

**Affiliations:** * National Institutes of Health, Bethesda, Maryland, USA;; † Armed Forces Institute of Pathology, Washington, DC, USA

**Keywords:** influenza, influenza epidemic, pandemic influenza

Figure 1

**Figure 1:**
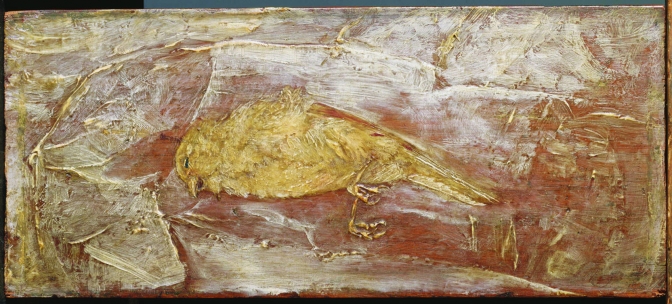
Dead Bird, by the influential 19th-century American artist Albert Pinkham Ryder (1847–1917), was first seen by Duncan Phillips no later than 1916 but was not purchased for the collection until it became available a decade later. The major scholarly catalog of The Phillips Collection, The Eye of Duncan Phillips: a Collection in the Making (*1*), calls Dead Bird "one of Ryder's most powerful images," noting that it "explores a recurrent illusory theme: the coexistence of the corporeal and the ethereal," and that "[s]uch starkly realistic details as the rigidly curled claws, rendered in heavy impasto, and the subtle textured contrasts of plumage and beak, create a moving evocation of suffering and death." - The Phillips Collection, Washington, DC. Reproduced with permission.

**Figure 2:**
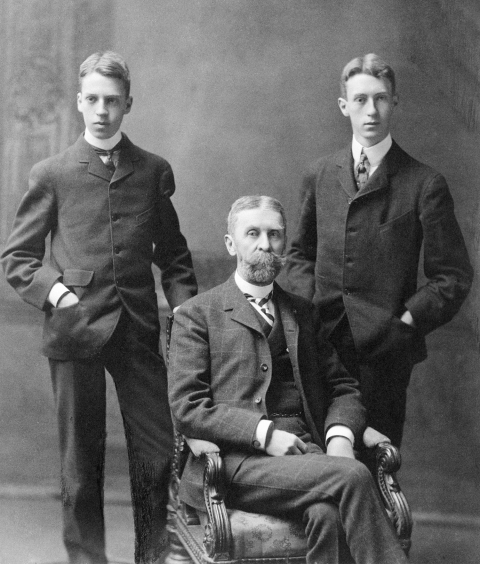
Duncan Clinch Phillips, Jr (left), his father Major Duncan Clinch Phillips (seated), and his brother James Laughlin Phillips, who died of influenza in October 1918. Photograph used with permission of The Phillips Collection, Washington, DC.

The two Phillips brothers (Figure 2) were so inseparable that when James, the older, was ready to leave home for Yale in 1902, he waited 2 years so that Duncan, the younger, could graduate from secondary school and accompany him. The brothers, who were full of energy and talent, spent their early years in Pittsburgh, where their maternal grandfather, James Laughlin Phillips, had achieved success as a banker and cofounder of the Jones and Laughlin Steel Company. Seeking a milder climate because of his health, the boys' father, Major Duncan Clinch Phillips, relocated the family to Washington, DC. In college, Duncan (the son) was elected an editor of the Yale Literary Magazine. Soon after college, James was appointed assistant treasurer of the Republican Party. Both developed a passionate love of contemporary art, and in 1916 their efforts to identify and purchase modern paintings had become so successful that James requested an annual stipend of $10,000 from their parents for the purchase of works of art for their growing collection.

But war had already broken out in Europe, and in 1917 the United States entered it. The brothers' patriotism overtook them, and they tried to enlist, even though they were pacifists at heart. Both were rejected for service. Duncan, turned down by both the Army and the Navy, was 30–40 pounds under the desired weight for his height, which suggested to recruiters the possibility of a chronic disease he in fact did not have. James had had prior bouts of pneumonia, and his military rejection may have been related to questions about his pulmonary status. Disappointed, James nonetheless arranged to marry his sweetheart Alice, with Duncan as best man.

But as was the case for so many in those dark years, the world was beginning to unravel. Their father died suddenly not long after the wedding. Surrounded by war and loss, James and Alice moved to Chevy Chase, Maryland, near the headquarters of the American Red Cross, where James became associate director of the Bureau of Personnel, in charge of applications for overseas war service. Then, in the fall of 1918, the "Spanish flu" struck James, and on October 21, he died in the family home in nearby Washington, DC. Her son's death broke the health of their mother, who became a semi-invalid. His secure world shattered, Duncan's health broke down too, and he gave in to despair.

"There came a time when sorrow all but overwhelmed me," he later wrote. "Then I turned to my love of painting for the will to live. Art offers two great gifts of emotion—the emotion of recognition and the emotion of escape. Both emotions take us out of the boundaries of self…. So in 1918 I incorporated the Phillips Memorial Gallery… to create a Memorial worthy of… my father… and my brother, James Laughlin Phillips, an idealist… a keen student of men and social conditions—a broad-minded, warm-hearted, lovable and very noble American" (*2*).

And so as a direct consequence of the death of his brother James from influenza, the 32-year-old Duncan Clinch Phillips, Jr (1886–1966) dedicated his life to creating a living memorial to him and to their father, and to establishing one of the finest public museums of modern art in the world. The collection, assembled over the next 5 decades, showed his remarkable taste, vision, and prescience in recognizing great works before others had suspected their greatness. Duncan's creative expression of feeling, the product of an artistic spirit, is reminiscent of similar creative expressions in literary form: the beautiful stories of Thomas Wolfe and Katherine Anne Porter, both of whom wrote about death and suffering from influenza. Wolfe's remarkable scene in Look Homeward, Angel (*3*) records the death of his own brother Benjamin from Spanish influenza, 2 days before the death of James Phillips. In Pale Horse, Pale Rider (*4*), Porter wrote a surrealistic but harrowing account of her own near death from influenza in 1918 and her belated discovery of the death from influenza of the lover who had cared for her. In each case, unbearable tragedy and loss were ennobled by art.

The collection assembled by Duncan Phillips and his wife Marjorie, herself a painter, focuses on modern art and its sources. The nearly 2,500 items included works by many now-famous 20th-century artists (van Gogh, Degas, Homer, Kandinsky, Klee, Matisse, O'Keeffe, Rothko) as well as earlier artists whose work Phillips believed anticipated modern art (Chardin, Goya, El Greco, Daumier). Phillips also championed many artists who were not well known at the time (Milton Avery, Pierre Bonnard, Karl Knaths, John Graham, Nicolas de Staël) and sometimes provided stipends to them (Arthur Dove, Augustus Vincent Tack).

Today The Phillips Collection is still housed in the family home, where James died, at 21st and Q Street, in northwest Washington, DC. The building itself is a work of architectural accomplishment, built in Georgian Revival style by Hornblower and Marshall in 1897. The paintings are exhibited in a warm intimate setting that encourages reflection and contemplation. Even though The Phillips Collection was conceived in sorrow and loss, Duncan Phillips wanted the viewing experience to be "joy-giving and life-enhancing" (*1*).
